# Estimating leaf area index of maize using UAV-based digital imagery and machine learning methods

**DOI:** 10.1038/s41598-022-20299-0

**Published:** 2022-09-24

**Authors:** Liping Du, Huan Yang, Xuan Song, Ning Wei, Caixia Yu, Weitong Wang, Yun Zhao

**Affiliations:** 1 School of Civil Engineering, Zhengzhou University, Zhengzhou, 450001 People’s Republic of China; 2 School of Water Conservancy Engineering, Zhengzhou University, Zhengzhou, 450001 People’s Republic of China; 3 School of Cyber Science and Engineering, Zhengzhou University, Zhengzhou, 450001 People’s Republic of China

**Keywords:** Agroecology, Environmental sciences

## Abstract

Leaf area index (LAI) is a fundamental indicator of crop growth status, timely and non-destructive estimation of LAI is of significant importance for precision agriculture. In this study, a multi-rotor UAV platform equipped with CMOS image sensors was used to capture maize canopy information, simultaneously, a total of 264 ground‐measured LAI data were collected during a 2-year field experiment. Linear regression (LR), backpropagation neural network (BPNN), and random forest (RF) algorithms were used to establish LAI estimation models, and their performances were evaluated through 500 repetitions of random sub-sampling, training, and testing. The results showed that RGB-based VIs derived from UAV digital images were strongly related to LAI, and the grain-filling stage (GS) of maize was identified as the optimal period for LAI estimation. The RF model performed best at both whole period and individual growth stages, with the highest R^2^ (0.71–0.88) and the lowest RMSE (0.12–0.25) on test datasets, followed by the BPNN model and LR models. In addition, a smaller 5–95% interval range of R^2^ and RMSE was observed in the RF model, which indicated that the RF model has good generalization ability and is able to produce reliable estimation results.

## Introduction

Leaf area index (LAI) refers to the total area of leaves per unit ground area, it is a key canopy structure parameter that is directly related to photosynthetic primary production, respiration, and evapotranspiration^1^. Maize is one of the most versatile crops in the world, it plays an important role in meeting the nutritional needs of millions of people and livestock production. The scientific and efficient estimation of LAI is of significance for the evaluation of maize growth potential, as well as providing reliable technical support for the optimization of field management practices^2^. Remote sensing (RS) technology offers means to monitor crop growth parameters in an effective, repetitive, and comparative way due to its non-invasive and high-flux characteristics^3^. The traditional satellite-based remote sensing images from MODIS, Landsat and SPOT5 with a spatial resolution of 1 km, 30 m, and 10 m have been widely applied in crop growth monitoring^4–6^. However, it is insufficient to attribute the dynamic change of LAI during the crop growth period due to the lengthy revisit intervals and the presence of clouds.

In recent years, unmanned aerial vehicles (UAV) have been increasingly used as an innovative remote sensing platform in agricultural fields^7^. In comparison to satellite remote sensing, the spatial and temporal resolution of UAV can be adjusted flexibly according to the requirements. This is particularly beneficial for crop monitoring with an short observation interval and cm-level spatial resolution^8,9^. Different sensors have been equipped on UAV for crop growth monitoring, of which the multispectral and hyperspectral sensors have shown great capability^10,11^. However, the high price and complex data processing process limited their popularization in agricultural observation, especially for developing countries with small-scale agriculture. Consequently, the UAV systems equipped with RGB cameras have received increasing attention due to their high spatial resolution and low cost.

Images captured by digital CMOS sensors contain not only red (R), green (G), and blue (B) bands of spectral information but also the spatial information of image pixels. The correlation between spectral features and crop LAI was commonly quantified by statistical regression models based on mono-temporal observation data^12–14^. Nevertheless, the spectral characteristics of crop canopy vary with growth stages, and the relationship between crop LAI and the image features is dynamic^15^. Thus, it is needed to accumulate sufficient information to establish models for characterizing crop LAI under different growth stages. In addition to this the non-linear relationship and multicollinearity could potentially exist between the spectral parameters and the LAI observed in multiple periods, whereas traditional statistical regression models have deficiencies when dealing with such complex relationships^16^.

With advancements in UAV and computer processing capacity, machine learning (ML) algorithms have boosted the power of obtaining relevant insights from the UAV-based agricultural remote sensing domain^17,18^. ML algorithms have unique advantages in modeling nonlinearity and heteroscedasticity relationships between the large amount of information contained in the images and crop growth parameters, which has been recognized as an effective approach for crop growth estimation^19^. Li et al. extracted the information from UAV-based images to estimate the LAI of rice by establishing different models, and random forest (RF) model exhibited the best predictive performance (R^2^ = 0.84)^20^. Azadbakht et al. evaluated the performance of different ML models in the inversion of wheat LAI^21^. Han et al. compared the performance of different ML methods to estimate the above-ground biomass of maize^22^. And Osco et al. predicted leaf nitrogen concentration and plant height with ML techniques and UAV-based multispectral imagery in maize^23^. These researches enriched the application of ML in UAV remote sensing, but the feasibility of combining ML and UAV-based RGB images for LAI estimation in maize during multiple growth stages has not been adequately investigated.

In this study, the UAV platform equipped with a CMOS sensor was used to capture maize canopy images through multiple growth stages. The traditional linear regression (LR) model, backpropagation neural network (BPNN), and random forest (RF) model were developed and compared for maize LAI estimation. The specific objectives of this study were: (1) explore the potential of UAV-based digital imagery for maize LAI monitoring; (2) evaluate the performance of different models and determine the optimal model for LAI estimation; (3) compare model performance in the whole growth period and individual growth stages; (4) indicate the optimal growth stage for maize LAI estimation.

## Materials and methods

### Experimental design

**Figure 1:**
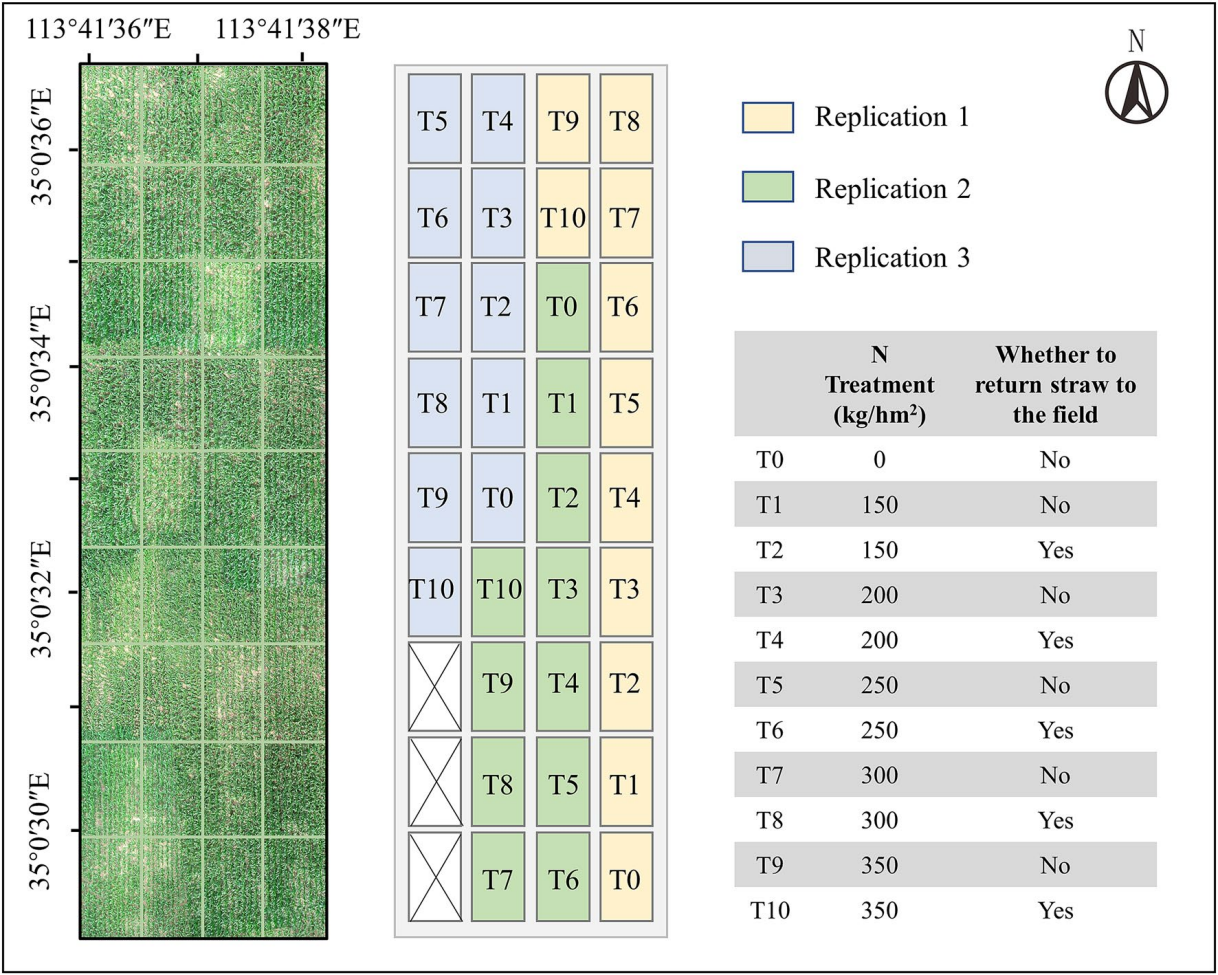
The experimental design.

A 2-year field experiment was conducted at the Modern Agricultural Research and Development Base of Henan Province (113° 35′–114° 15′ E, 34° 53′–35° 11′ N). In order to enhance the diversity of LAI data, a split-plot design with a variety of field management measures and three replications was selected for the experiment (Fig. 1). The size of each experiment plot was 40 m^2^, the soil texture was predominantly sandy loam and sandy clay loam, as determined by textural analysis of soil samples collected before planting. Maize cultivar Dedan-5 was used in the experiment, which was planted on June 12, 2019, and June 20, 2020, with a row spacing of 42 cm and a planting density of 7 seedlings·m^−2^. The soil and cultivar in field experiments were representatives of those in the region. The irrigation, pesticide, and herbicide control practices followed local management for maize production.

### LAI measurements and UAV-based image acquisition

The measurements of LAI were conducted at four growth stages including the tasseling stage (TS), flowering stage (FS), grain-filling stage (GS), and milk-ripe stage (MS) of maize in 2019 and 2020, a total of 264 LAI data of maize were collected during the 2-year field trial (Table 1). In order to reduce the impact of plant variability, the random sampling method was used to collect LAI samples. For each plot, three plants were randomly selected to measure the total green leaf area with the non-destructive portable leaf area meter (Laser Area Meter CI-203; CID Inc.). And the average leaf area of selected plants represented the single plant leaf area in each experiment plot. The LAI of each plot was

**Table 1:** Description of samplings.

Year	Planting date	Sensing and sampling date	Number of samples
2019	June 12	30 July (TS)	33
		6 August (FS)	33
		13 August (GS)	33
		19 August (MS)	33
2020	June 20	9 August (TS)	33
		17 August (FS)	33
		26 August (GS)	33
		3 September (MS)	33

1 $$\mathrm{LAI}=\mathrm{LA}*\mathrm{D}$$

where $\mathrm{LA}$ is the leaf area of a single plant in each plot; $\mathrm{D}$ is the planting density in one square meter.

PHANTOM 4 PRO (DJI-Innovations Inc., Shenzhen, China) is a multi-rotor UAV equipped with a 20-megapixel visible-light camera that was employed to capture digital images. Aerial observations were conducted on the same dates as the LAI measurements, which was between 10:30 a.m. and 2:00 p.m. local time when the solar zenith angle was minimal. The UAV was flown automatically based on preset flight parameters and waypoints, with a forward overlap of 80% and a side overlap of 60%. A three-axis gimbal integrated with the inertial navigation system stabilized the camera, the automatic camera mode with fixed ISO (100) and a fixed exposure was used during the flight. Altogether, 4192 images were taken in eight flights from a flight height of 29.36 m above ground, with a spatial resolution of 0.008 m.

The measurements of maize LAI were carried out with permission from the Modern Agricultural Research and Development Base of Henan Province. All experiments were carried out in accordance with relevant institutional, national, and international guidelines and legislation.

### Image pre-processing

DJI Terra (version 2.3.3) was used to generate ortho-rectified images based on the structure from motion algorithms and a mosaic blending model. The main procedures are as follows: (1) extract feature points and match features according to the longitude, latitude, elevation, roll angle, pitch angle, and heading angle of each image; (2) build dense 3D point clouds by using dense multi-view stereo matching algorithm; (3) build a 3D polygonal mesh based on the vector relationship between each point in the dense cloud; (4) establish a 3D model with both external image and internal structure by merging the mosaic image into the 3D model; (5) generate digital orthophoto map (DOM).

### Vegetation indices (VIs) derived from the UAV-based digital imagery

Digital imagery records the intensity of visible red (R), green (G), and blue (B) bands in individual pixels^24^. In order to enhance the vegetation parameters contained in the digital image, fourteen commonly used RGB-based VIs were collected, and their correlation with the LAI of maize at different growth stages was evaluated. Table 2 shows the detailed information of the selected RGB-based VIs.

**Table 2:** RGB-based VIs for LAI estimation.

VIs	Full name	Formula	References
BRRI	Blue-Red Ratio Index	$b/r$	^25^
RGRI	Red Green Ratio Index	$r/g$	^26^
BGRI	Blue Green Ratio Index	$b/g$	^27^
NGRDI	Normalized Green–Red Difference Index	$(g-r)/(g+r)$	^28^
NGBDI	Normalized Green–Blue Difference Index	$(g-b)/(g+b)$	^29^
EXR	Excess Red Vegetation Index	$1.4r-g$	^30^
EXG	Excess Green Vegetation Index	$2g-r-b$	^31^
EXB	Excess Blue Vegetation Index	$1.4b-g$	^32^
EXGR	Excess Green minus Excess Red Vegetation Index	$EXG-EXR$	^33^
CIVE	Color index of vegetation	$0.44r-0.88g+0.39b+18.79$	^34^
VARI	Visible Atmospherically Resistant Index	$(g-r)/(g+r+b)$	^28^
MGRVI	Modified Green Red Vegetation Index	$({g}^{2}-{r}^{2})/({g}^{2}+{r}^{2})$	^35^
RGBVI	Red Green Blue Vegetation Index	$({g}^{2}-b\times r)/( {g}^{2}+b\times r)$	^36^
VDVI	Visible-band Difference Vegetation Index	$(2g-r-b)/(2g+r+b)$	^37^

Centered on the point where LAI was measured, regions of interests (ROIs) with a size of 100*100 were clipped from the digital image. Python 3.7.3 was used for extracting the R, G, B information of maize and computing the RGB-based VIs from ROIs. In order to reduce the effects of light and shadow, the R, G, B color space of the image was normalized according to the followings:

2 $$\mathrm{r}=\frac{R}{R+G+B}$$

3 $$g=\frac{G}{R+G+B}$$

4 $$b=\frac{B}{R+G+B}$$

where r, g, and b are the normalized values. R, G, B are the pixel values from the digital images based on each band.

### Pearson correlation analysis

Before regression analysis, the Pearson correlation analysis was performed to determine the relationship between maize LAI and different RGB-based VIs extracted from the digital image. Pearson correlation coefficient ($\mathrm{r}$) reflects the degree of linear correlation between two variables, which is between − 1 and 1. The calculation formula of Pearson correlation coefficient was expressed as follows:

5 $$\mathrm{r}= \frac{\sum_{i=1}^{n}\left({X}_{i}-\overline{X }\right)\left({Y}_{i}-\overline{Y }\right)}{\sqrt{\sum_{i=1}^{n}{\left({X}_{i}-\overline{X }\right)}^{2}}\sqrt{\sum_{i=1}^{n}{\left({Y}_{i}-\overline{Y }\right)}^{2}}}$$

where $X$, $\mathrm{Y}$ are variables, $n$ is the number of variables.

### Regression methods

#### Linear regression (LR)

Linear regression is an approach for modelling the relationship between dependent and independent variables. The case of one independent variable is called unary linear regression (ULR), the expressions can be expressed as follows:

6 $$\mathrm{y}={\beta }_{0}+{\beta }_{1}x+\varepsilon $$

where $\varepsilon$ is deviation, which satisfies the normal distribution. $x$, $\mathrm{y}$ are variables. ${\beta }_{0}$, ${\beta }_{1}$ are the intercept and slope of the regression line, respectively.

For more than one independent variable, the regression process is called multiple linear regression (MLR), the expressions can be expressed as:

7 $$\mathrm{y}={\beta }_{0}+{\beta }_{1}{x}_{1}+{\beta }_{2}{x}_{2}+\dots +{\beta }_{n}{x}_{n}$$

where $${x}_{1}$$,$$ {x}_{2}$$, …, $${x}_{n}$$, $$\mathrm{y}$$ are variables, $${\beta }_{0}$$, $${\beta }_{1}$$, $${\beta }_{2}$$, …, $${\beta }_{n}$$ are coefficients that determined by least square method and gradient descent method^38^.

The RGB-based VIs with the highest Pearson correlation coefficient was used to establish the ULR model, and VIs with a correlation coefficient higher than 0.7 were used to establish the MLR model. In each growth stage, 70% of observation data were randomly selected for establishing models, and the remaining 30% of data were used as the testing dataset to assess the model performance.

#### Back propagation neural networks (BPNN)

**Figure 2:**
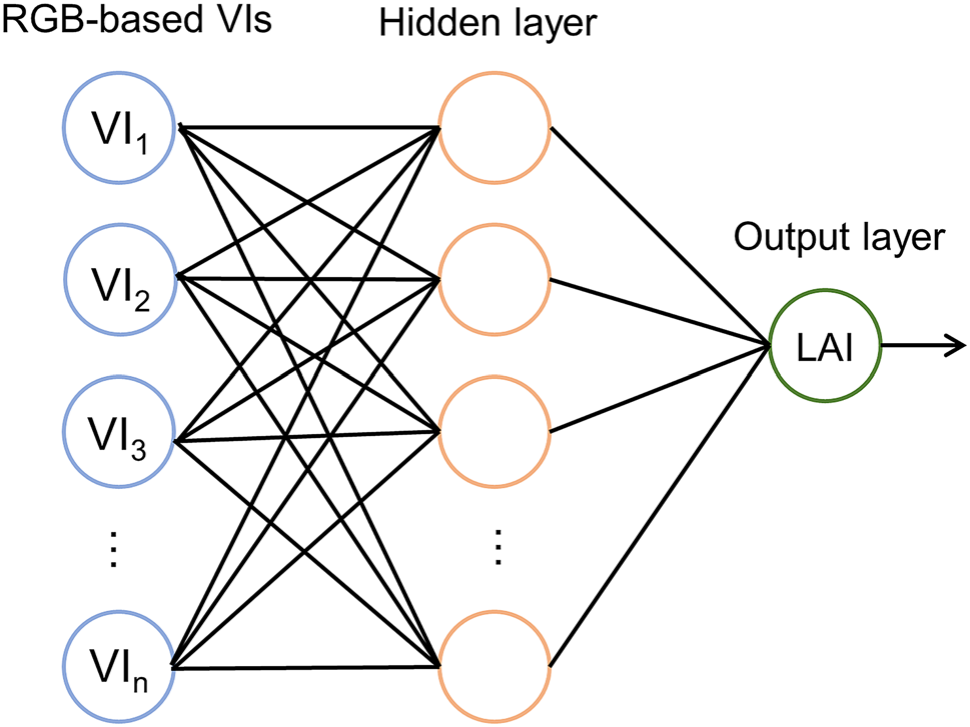
Three-layer BPNN model.

In this study, a three-layer BPNN model was established for LAI estimation (Fig. 2). RGB-based VIs with a correlation coefficient higher than 0.7 were selected as the input variables. Tan-Sigmoid activation function was used in the hidden layer, and the Levenberg–Marquardt algorithm was selected as the training function. The maximum epoch of BPNN training was set to 1000, the learning rate was set to 0.005, and the MSE was set to 0.001. The observation data set was split into the training set and the testing dataset with a ratio of 7:3. The training dataset was used to fit the weights and bias of the BPNN model, the testing dataset was used to evaluate the model performance. Before training, data normalization was conducted for the input and output variables, and the denormalization was required to convent the output variable back into the original units after training.

#### Random forest (RF)

**Figure 3:**
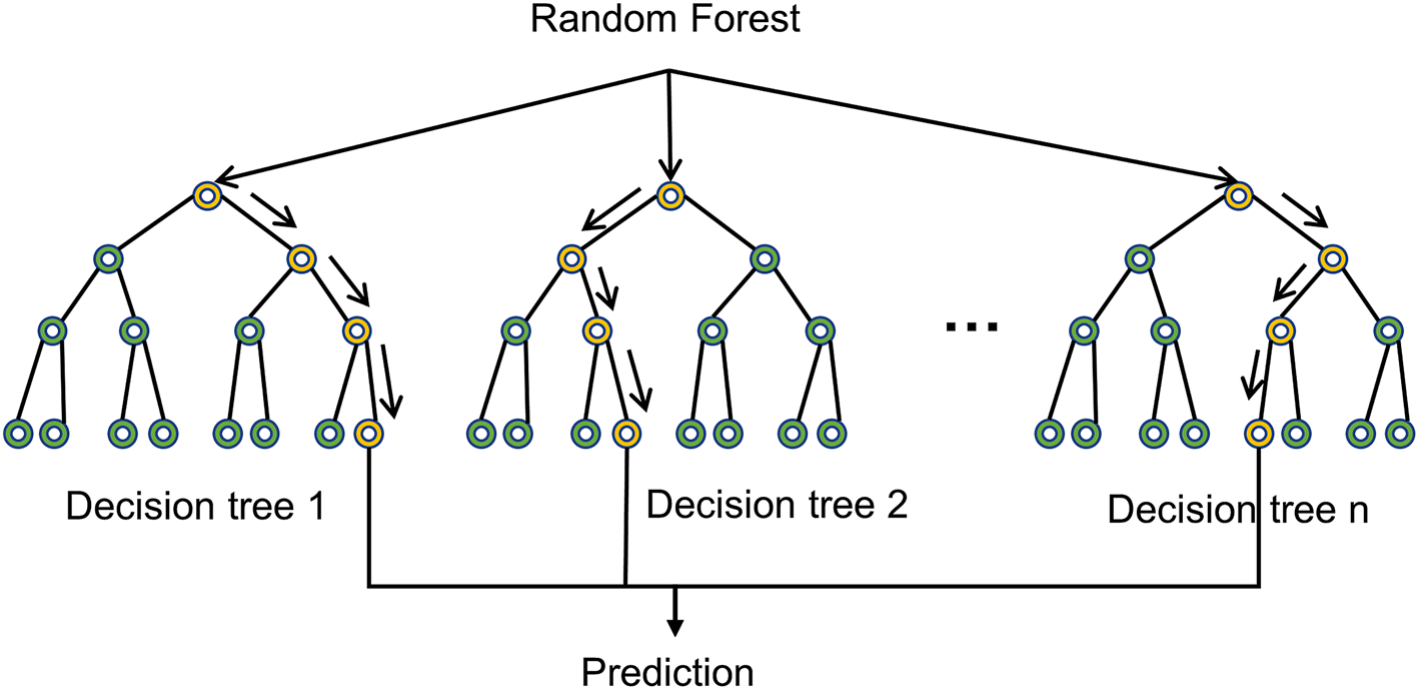
Random forest model.

RF is a non-parametric ensemble ML method that operates by constructing a multitude of decision trees at training time and outputting the average prediction of the individual trees (Fig. 3). The bootstrapping approach was used to collect different sub-training data from the input training dataset to construct individual decision trees.

The construction process of RF regression model is as follows:

1. The value of $\mathrm{n}\_\mathrm{estimators}$ was tested from 50 to 1000 in increments of 50, and the value of 500 was finally selected according to higher R^2^ and lower RMSE.
2. At each node per tree, $\mathrm{m}\_\mathrm{try}$ RGB-based VIs was randomly selected from all 14 vegetation indices, and the best split was chosen according the lowest Gini Index. $\mathrm{m}\_\mathrm{try}$ was tested from 3 to 10, and the final value was 6.
3. The other parameters in the RF model were kept as default values according to the $\mathrm{RandomForestRegressor}$ function in $\mathrm{Scikit}-\mathrm{learn library}$.
4. For each tree, the data splitting process in each internal node was repeated from the root node until a pre-defined stop condition was reached.
5. Similar with LR and BPNN model, the RGB-based VIs with a correlation coefficient higher than 0.7 were selected as the input variables, and the output variable is LAI.

### Data analysis and performance evaluation

**Figure 4:**
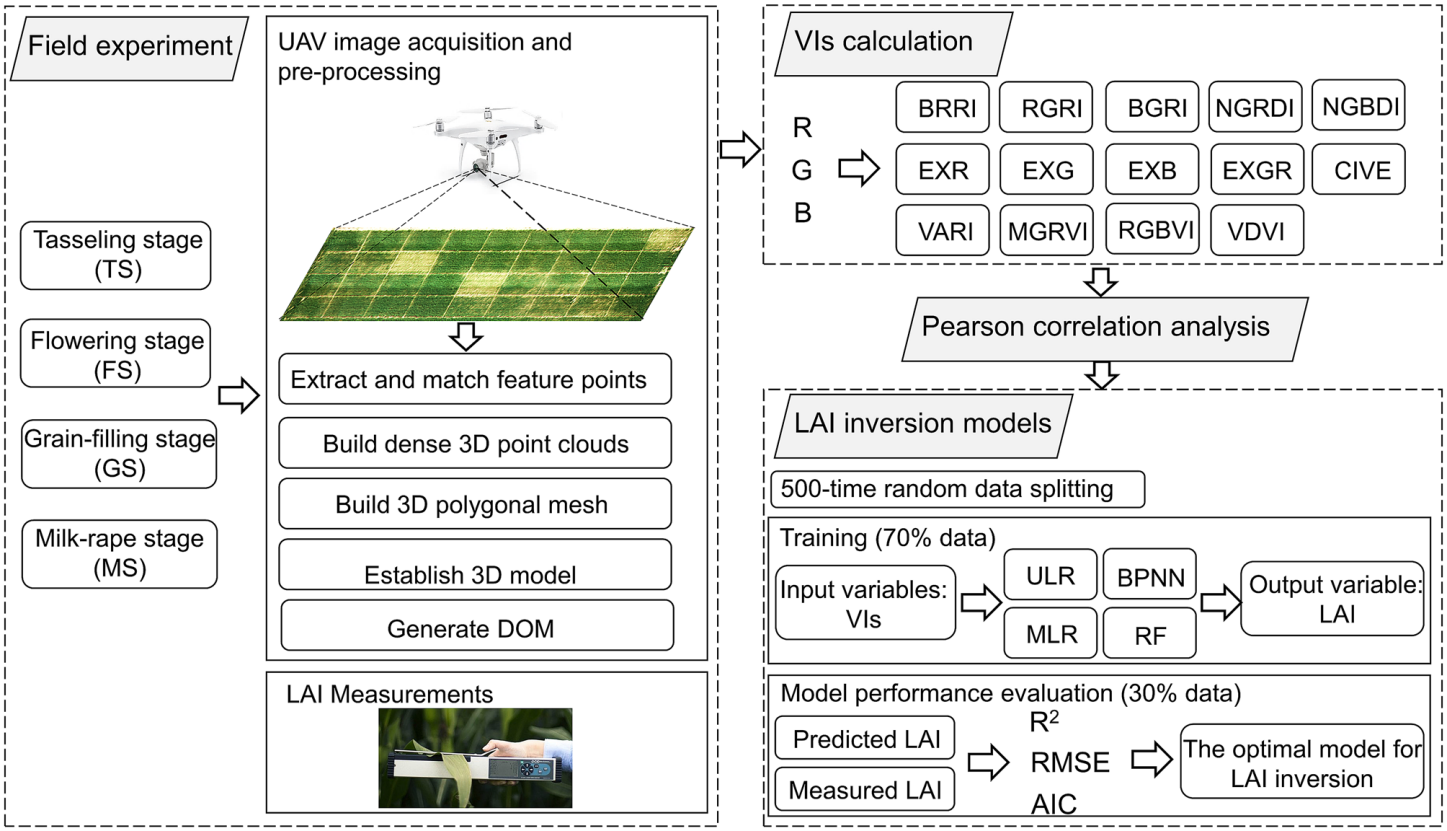
Flowchart of LAI inversion using UAV-based remote sensing and ML methods.

The repeated random sampling validation method was used to evaluate the generalization performance of different models. The training and testing dataset were randomly split 500 times. For each split, the LR, BPNN, and RF models were fitted to the training dataset, and the estimation accuracy was evaluated using the testing dataset. The coefficient of determination (R^2^), root mean square error (RMSE), and Akaike information criterion (AIC) of the training dataset were used for the assessment of models^39^, and the estimation accuracy was evaluated by R^2^ and RMSE of the testing dataset. Mathematically, a higher R^2^ corresponds to a smaller RMSE, and thus represents better model performance. The procedures of LAI inversion using UAV-based digital imagery and ML methods were shown in Fig. 4.

The construction and evaluation of models was performed using Python 3.7.3 in Windows 10 operating system with Intel Core i7-9700 processor, 3.00 GHz CPU, and 32 GB RAM. The processing software is Spyder. The statistical analysis and figure plotting were performed in R × 64 4.0.3.

## Results

### Descriptive statistics

Table 3 shows the descriptive statistics of the measured LAI covering four growth stages, eleven treatments, and 2 years. The results indicated that there were significant differences in the LAI during the 2-year field experiment. The LAI at individual growth stages ranged from 1.98 to 4.51 with high coefficients of variation (7.68–15.72) in terms of the wide range of N treatments and different straw returning measures. Furthermore, the large variability of LAI was supposed to cover most of the possible situations, which also provides convincible datasets for the applicability of UAV-based digital imagery for maize LAI estimation.

**Table 3:** Descriptive statistics of the measured LAI.

Growth stage	Samples	Min	Max	Mean	Standard deviation	Coefficient of variation (%)
TS	66	1.98	3.90	2.89	0.45	15.72
FS	66	2.76	4.31	3.62	0.39	10.71
GS	66	3.20	4.51	3.95	0.30	7.68
MS	66	2.43	4.08	3.41	0.36	10.55
ALL	264	1.98	4.51	3.47	0.54	15.60

### Correlation of the RGB-based VIs and LAI

**Figure 5:**
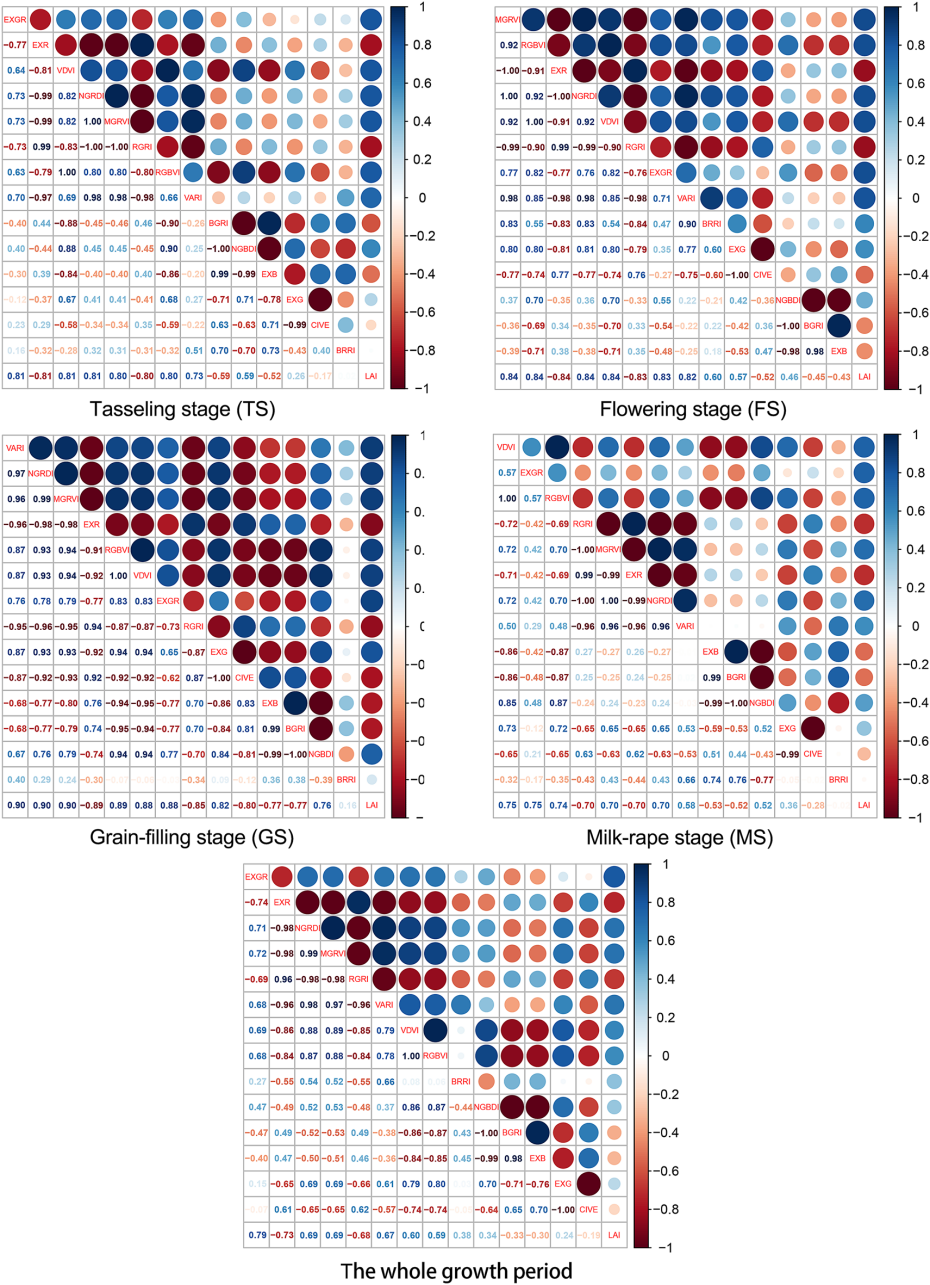
Pearson correlation coefficients between RGB-based VIs and LAI.

Figure 5 shows the Pearson’s correlation coefficients between RGB-based VIs and the LAI of maize. The results indicated that most RGB-based VIs including RGRI, NGRDI, EXR, EXGR, MGRVI, RGBVI, and VDVI had significant correlations with LAI at different growth stages. The maximum correlation was observed at GS, with the highest correlation coefficient of 0.90 (VARI, MGRVI). The correlations were also strong at TS and FS, with the highest correlation coefficients of 0.81 and 0.84, respectively. While the correlation between RGB-based VIs and LAI decreased at MS (EXGR: 0.75; VDVI: 0.75; RGBVI: 0.74). Generally, there was a strong correlation between RGB-based VIs and the LAI of maize at individual growth stages, but the correlation was much weaker with regard to the whole growth period (EXGR: 0.79; EXR: 0.73; NGRDI: 0.69; MGRVI: 0.69).

### LAI estimation performance in the whole growth period

**Figure 6:**
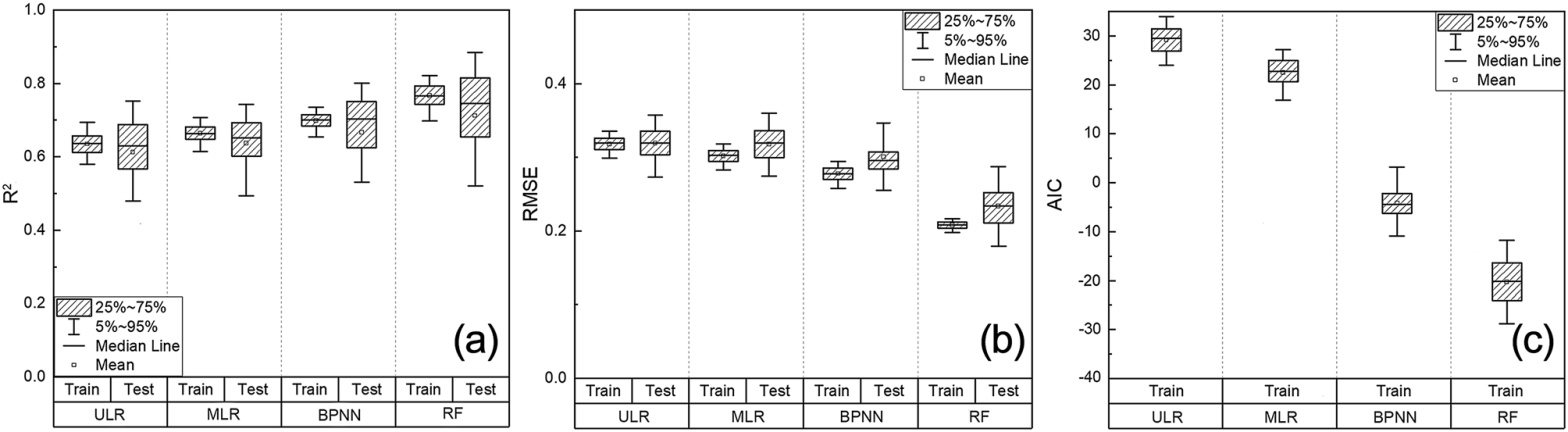
Performances of different regression models for LAI estimation in the whole growth period (**a** R^2^ of different regression models. **b** RMSE of different regression models. **c** AIC of different regression models.).

The performances of different regression models for maize LAI estimation were explored through 500 times data splitting, training, and testing (Fig. 6). Results revealed that the performance of the MLR model (Training: R^2^ = 0.66, RMSE = 0.30, AIC = 22.43; Testing: R^2^ = 0.64, RMSE = 0.32) with multiple RGB-based VIs as input variables was better than that of the ULR model (Training: R^2^ = 0.63, RMSE = 0.32, AIC = 29.33; Testing: R^2^ = 0.63, RMSE = 0.32). The average R^2^ of the BPNN and RF models was 0.71 and 0.77, respectively in the training dataset, and the RF model has higher accuracy and lower complexity (RMSE = 0.21, AIC = − 20.2). In addition to this, the RF model had better performance in the testing dataset with an average R^2^ of 0.71 and an average RMSE of 0.25 compared with the BPNN model (R^2^ = 0.67; RMSE = 0.30).

**Figure 7:**
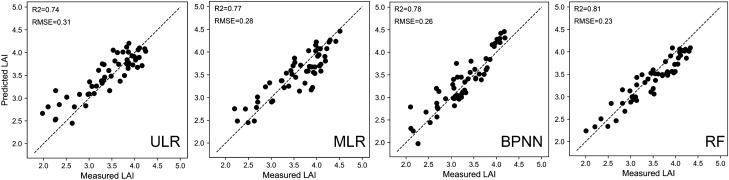
Comparison between the predicted LAI and measured LAI with different regression models.

Figure 7 shows the predicted and measured LAI of each regression model. The result suggests that the data points are generally close to the 1:1 line, especially when using the BPNN and the RF models. The performances of the LR models were inferior to the BPNN and the RF models due to the complex relationships between the RGB-based VIs and measured LAI data during the entirety of the growth period. It was further observed that a small portion of data points associated with low values of measured LAI was located above the diagonal for all the models, which suggested that the LAI may be overestimated with digital imagery in the case of small leaf area of maize.

### LAI estimation performance at individual growth stages

**Figure 8:**
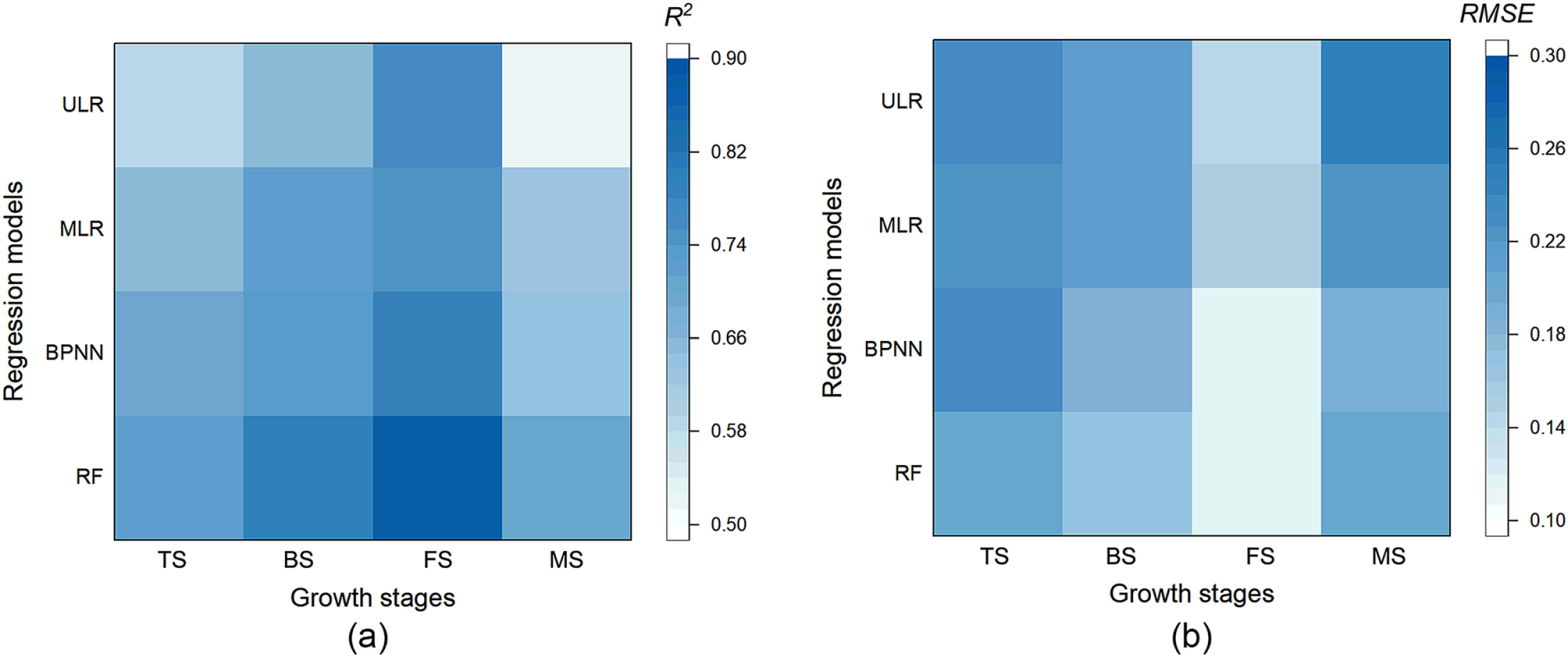
Comparison of LAI estimation models at different growth stages (**a** average R^2^ of different regression models. **b** Average RMSE of different regression models.).

Figure 8 indicated that the performance of LAI estimation with UAV-based digital imagery varied with growth stages. GS was the optimal growth stage for LAI estimation with R^2^ ranging from 0.75 to 0.88, and RMSE ranging from 0.12 to 0.15, followed by FS (R^2^: 0.65–0.8, RMSE: 0.17–0.21) and TS (R^2^: 0.59–0.72, RMSE: 0.20–0.23), the lowest estimation accuracy was observed in MS (R^2^: 0.52–0.71, RMSE:0.19–0.25) since part of the leaves of maize was withered during this period, which resulted in a significant increase of soil pixels in the digital image, and affects the value of RGB-based VIs.

**Figure 9:**
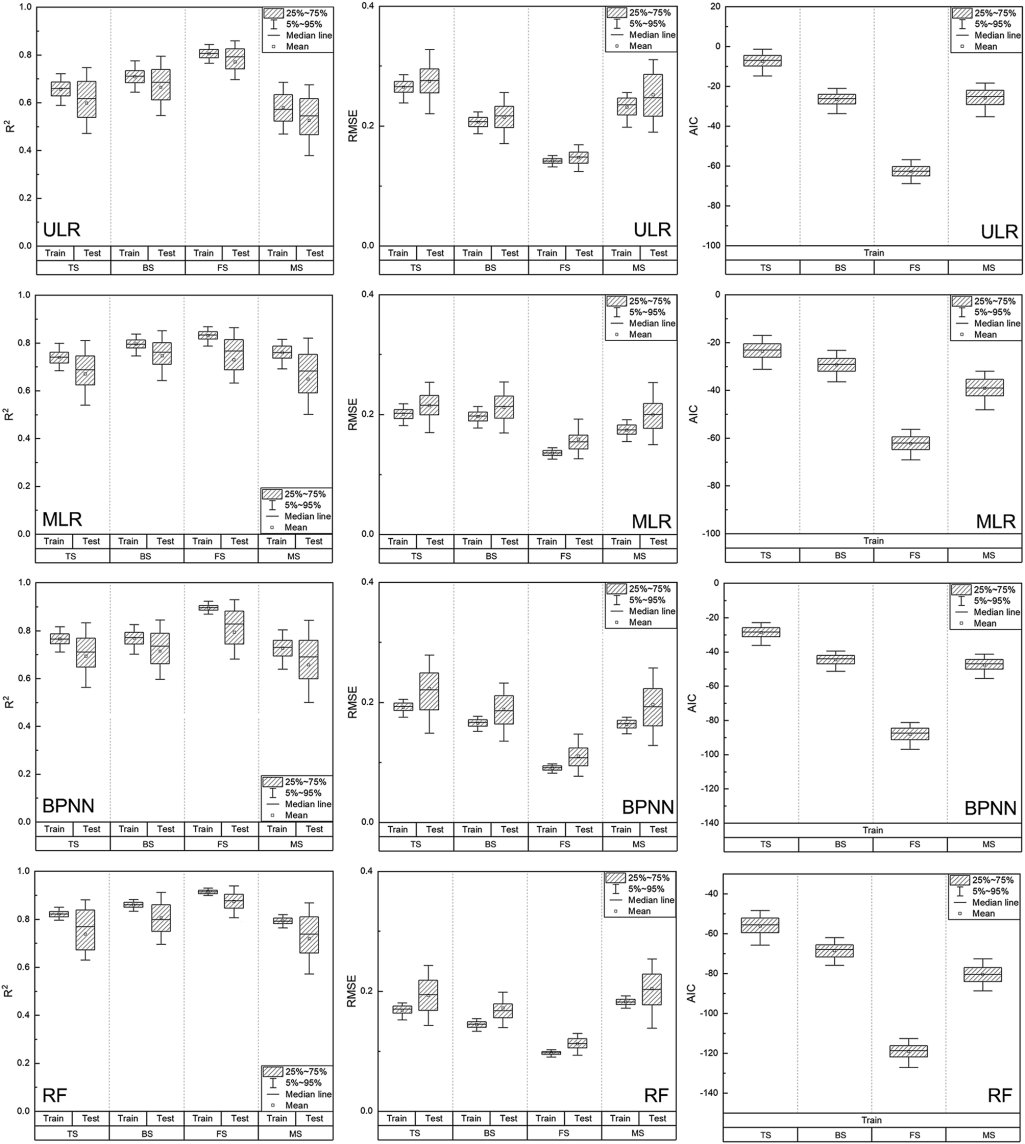
Performances of different regression models for LAI estimation at individual growth stages (Left. R^2^ of different regression models. Middle. RMSE of different regression models. Right. AIC of different regression models.).

The R^2^, RMSE, and AIC of different regression models for maize LAI estimation at individual growth stages were shown in Fig. 9. It was observed that the R^2^ increased, whereas the RMSE and AIC decreased with the increase in the number of input variables in LR models, especially at the TS and MS. There was little difference in R^2^ between the BPNN model and the MLR model in each growth stage, but the RMSE and AIC of the BPNN model were much lower than the MLR model, which suggests that the BPNN model had higher accuracy for LAI estimation, and could interpret data with fewer parameters.

Compared with the LR and the BPNN models, the RF model was considered as the best performing model at individual growth stages with higher R^2^ and lower RMSE on both training and testing datasets. At GS, the average R^2^ of the RF model reached 0.90 on the training dataset and 0.88 on the testing dataset, with RMSE at the lowest levels. The RF model also showed good performance even at MS with poor data quality (R^2^ = 0.72, RMSE = 0.20).

**Figure 10:**
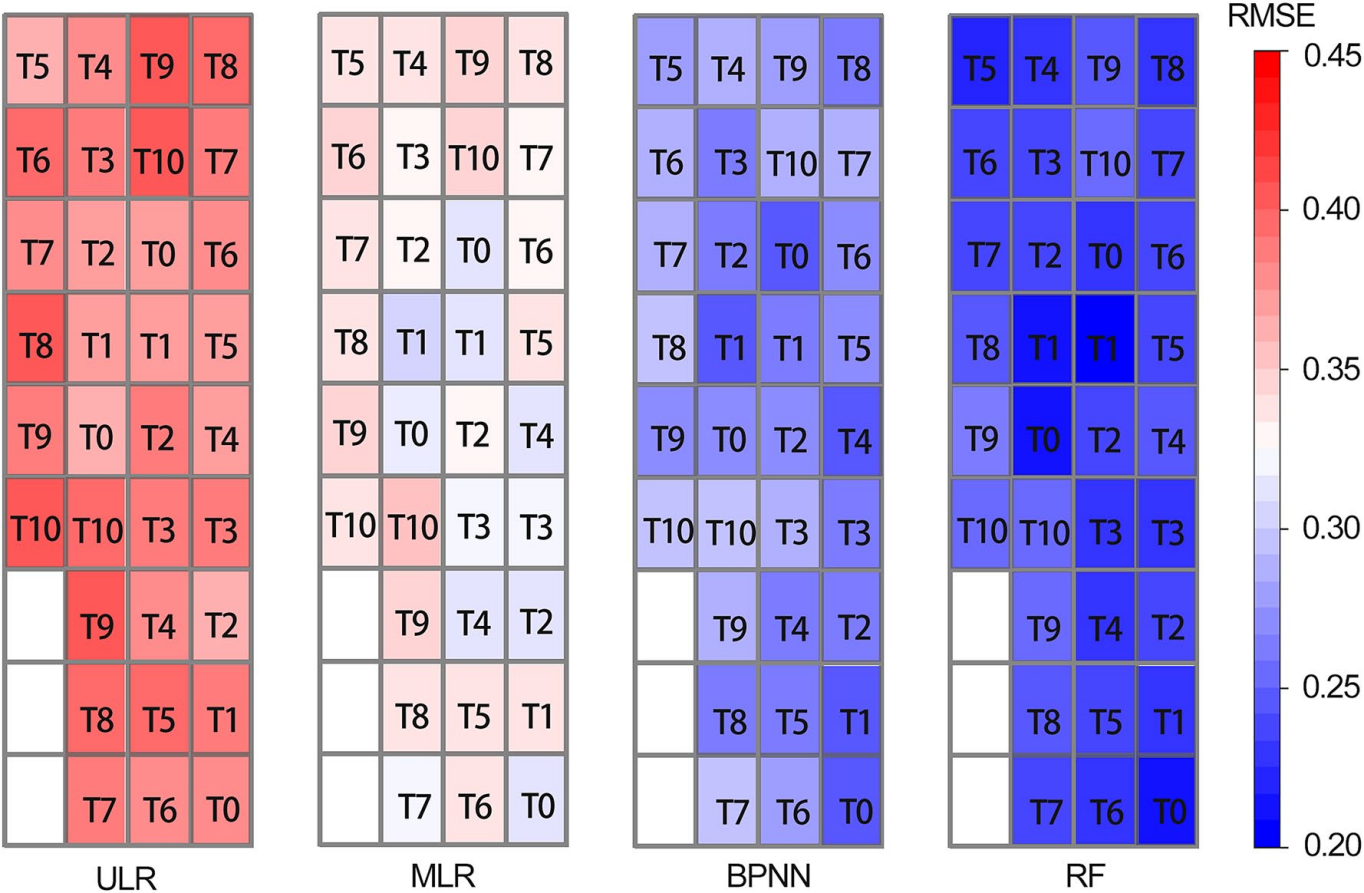
RMSE between the predicted LAI and the measured values for the whole experimental area.

Taking the GS stage as an example, the predicted LAI of the whole experimental area was compared with the measured value based on UAV-based digital imagery to further explore the application potential of the proposed LAI estimation models. Figure 10 shows the RMSE between the predicted LAI of different models and the measured values of each experimental plot. Generally, the ML algorithms used in the study can accurately simulate the LAI under different field treatment measures. It is confirmed that the RF model has the highest LAI estimation accuracy, with RMSE between 0.20 and 0.26 for the whole area, while the RMSE of the ULR model and MLR model was relatively low. In addition, it is noticed that plots with high N application usually had relatively lower LAI estimation accuracy. For instance, the RMSE of T9 and T10 were more than 0.3 higher than that of T1 and T2. This is due to the fact that in plots with high N application, vegetation cover is usually high and RGB-based VIs calculated based on UAV images tend to be saturated, which increases the estimation error of LAI^40,41^.

## Discussion

### The linkage between UAV-based digital imagery and LAI

UAV-based digital imagery has been recognized as a prospective approach for agricultural machine vision applications due to its low cost, large quantities of information volume, and maneuverability. However, it is insufficient to use the original spectral feature of digital sensors for quantitative analysis of crop phenotypic information because the digital imagery contains only three bands of spectral information. The RGB-based VIs, obtained by diverse spectral transformation of visible bands, have been recognized as an effective way to enhance the crop spectral features that have been widely used in the extraction of crop canopy information^42^. In this study, fourteen RGB-based VIs, which are most frequently referred to in the previous research, were collected to explore their correlation relationship with the LAI of maize. It was observed that RGRI, NGRDI, EXR, EXGR, MGRVI, RGBVI, and VDVI were strongly related to LAI at all growth stages, which indicates that there is a great potential for establishing maize LAI estimation models with RGB-based VIs derived from digital images. Similar results were also found in wheat LAI prediction with a digital orthophoto map^43^.

### Comparison of different regression models

In this study, we mitigated the impact of data splitting on model performance through a 500-time repeated random sub-sampling and evaluated the accuracy and robustness of the LR model, the BPNN model, and the RF model. The LR model is the most basic regression model in the field of ML^44^, and it can be divided into the ULR model and MLR model according to the number of input variables. The results suggested that the LR model had the lowest accuracy in maize LAI estimation, which is consistent with the results of the study by Li et al.^20^. The performance of the MLR model was better than that of the ULR model. This is attributed to the increasing number of input RGB-based VIs, which allow more spectral information to be used for modeling, therefore making up for the deficiency of spectral information saturation caused by a single input variable^41^. It is worth noting that the improvement of model performance was more obvious at the MS when the number of soil background pixels increased in the UAV-based digital image. From this, it can be inferred that the combination of multiple RGB-based VIs could reduce the influence of environmental background in the UAV-based digital imagery to a certain extent. In general, the performances of both the ULR and the MLR models were relatively poor compared with the BPNN and the RF models, which indicates the potential applicability of non-linear structures for LAI estimation^22^.

BPNN is a powerful ML algorithm that has the advantages of strong non-linearity and self-organization ability when dealing with complex and nonlinear approximation problems^45,46^. Compared with the LR models, the three-layer BPNN model used in this study has significantly improved the LAI estimation performance in the whole growth period. However, Fig. 7 indicated that there was only a slight improvement in the average R^2^ and RMSE for individual growth stages and, the 5–95% range of R^2^ and RMSE on the testing dataset showed apparent extension through multiple times of dataset split. This phenomenon is predominantly due to the strong sample dependencies of the BPNN model^47^. The performance of the BPNN model is closely related to the typicality of training samples. In the entire growth stage, more sample features could be used for training at each dataset split, which guarantee a higher stability of model performance. Regarding the individual growth stages, the smaller data size makes the extracted training data unable to fully reflect the general rule and weakens the stability of the BPNN model. Therefore, the BPNN model could not exhibit better performance for LAI estimation than the MLR model when the data size is small, which is consistent with the inference proposed by^48^.

The RF model showed the best performance for LAI estimation with the highest R^2^ and lowest RMSE, and the 25–75% and 5–95% interval ranges of each evaluation index were narrowed down in both the whole growth period and each growth stage. This indicated the RF model has strong stability and generalization ability in LAI estimation. The result was in agreement with the studies reported by Zha et al.^49^ in rice nitrogen nutrition estimation. The superior estimation performance of the RF model is mainly attributed to the numerous independent, complex and powerful learners, which makes the algorithm more robust to the noises contained in the variables, and results in high model accuracy regardless of the data splitting^48^. Besides, the random selection of variables and the bootstrap aggregating algorithm in the RF model can efficiently reduce the autocorrelation of input RGB-based VIs as well as avoid over-fitting^50,51^.

### The optimal growth stage for maize LAI estimation

The ability to rapidly estimate crop LAI in real-time based on UAV-based imagery is a promising area of potential research. In this study, multiple times field observations were carried out at four typical growth stages of maize including TS, FS, GS, and MS. At each growth stage, the relationship between LAI and RGB-based VIs was investigated, and the estimation accuracy of different algorithms was analyzed. Results suggested that the RGB-based VIs of the GS, FS, and TS had higher correlations with the LAI of maize, while the correlation at MS was relatively poor. Besides, the LAI estimation performance of different regression models varied with the change of growth stages, the highest estimation accuracy was observed at GS, followed by FS, TS, and MS. These findings indicated that RGB-based VIs derived from UAV imagery performed well in LAI estimation in the middle growth stages of maize.

### Implications for future work

The result of this study showed the feasibility of the UAV-based digital imagery and ML algorithms for maize LAI estimation at both the whole growth period and individual growth stages. To improve the diversity and detail of select datasets, a 2-year field experiment in a split plot with different nitrogen treatments and straw returning measures was conducted. Additionally, a loop was added in each algorithm to randomly split the dataset many times in order to reduce the impact of data splitting on estimation errors, as well as test the stability of models. It is expected that the combination of UAV-based digital imagery and ML methods could be transferred to different crop types as well as different crop growth parameters such as plant height, above-ground biomass, and yield. Furthermore, the UAV-based digital image used in this study has not been processed by pixel segmentation, which may have a negative impact on the estimation accuracy of LAI, especially at certain growth stages. It is also an important part that needs to be explored in further research.

For traditional ML, feature extraction is generally completed manually, it is laborious and requires experience and professional knowledge, therefore, deep learning is becoming a hot subject since it could let the computer automatically learn features from images and eliminates the complex manual information extraction process. But the UAV-based digital imagery has a centimeter-level resolution and carries a large amount of information, it requires plenty of computing resources to complete training of a deep learning model. In the next step of research, we will seek the support of the cloud computing platform, the convolutional neural network (CNN), a typical deep learning algorithm, is going to be implemented in the estimation of LAI based on UAV-based imagery, and the results will be compared and discussed with those of this study.

## Conclusions

This study demonstrated the feasibility of UAV-based digital images and ML algorithms for maize LAI estimation through a 2-year field trial. The RGB-based VIs including RGRI, NGRDI, EXR, EXGR, MGRVI, RGBVI, and VDVI derived from digital images had significant correlations with LAI of maize at individual growth stages, but the correlation was weaker with regard to the whole growth period. GS was the optimal stage for LAI estimation, followed by FS, TS, and MS. The RF model showed the highest accuracy for LAI estimation with an average R^2^ of 0.71, RMSE of 0.25 for the whole growth period, and R^2^ ranged from 0.71 to 0.88, RMSE ranged from 0.12 to 0.2 for individual growth stages. The smaller 5–95% interval range of R^2^ and RMSE of the RF model suggested that the model was less affected by dataset size and outliers compared with other ML algorithms, which indicated that the combination of UAV-based digital imagery and the RF method is a promising way for timely and accurately monitoring LAI with high spatial resolution. Furthermore, this approach may offer a theoretical framework that could be transferred to different crop types as well as different crop growth parameters.

## Data Availability

The datasets used during the current study available from the corresponding author on reasonable request.
